# Supplementary material for: Comparative multiomics analysis of cell physiological state after culture in a basket bioreactor
Source: Sci Rep. 2022 Nov 23;12:20161. doi: 10.1038/s41598-022-24687-4 (PMC9686226; doi:10.1038/s41598-022-24687-4)
Supplement: Supplementary file 1 — Supplementary Information 1. [file 41598_2022_24687_MOESM1_ESM.zip › raw data/Transcriptomic raw data/6.Enrichment/1.GO/TE1vsTC1/UP/TE1vsTC1.up.GOcc_DAG.pdf]

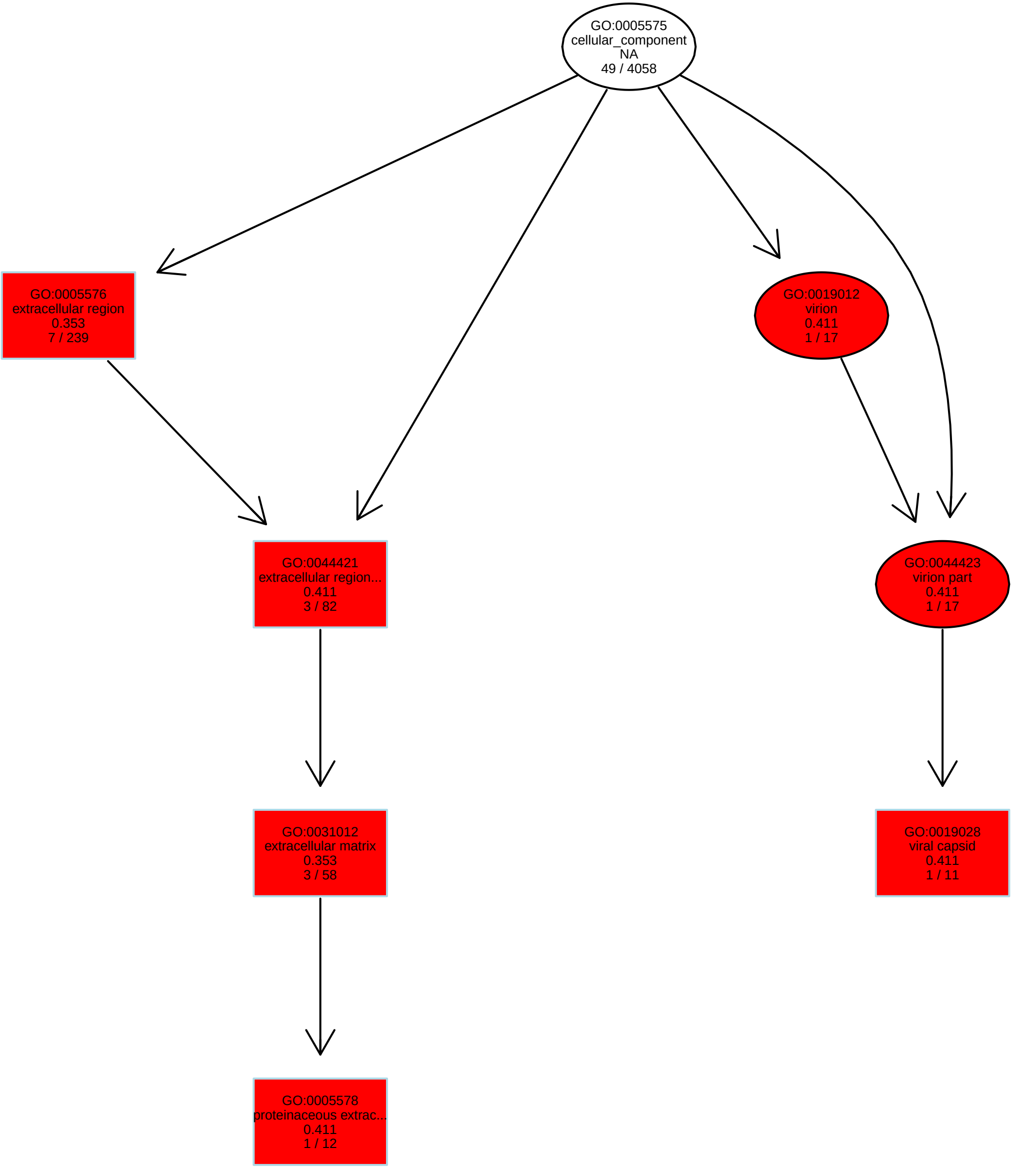
GO:0005575
cellular_component
NA
49 / 4058
GO:0005576
extracellular region
0.353
7 / 239
GO:0019012
virion
0.411
1 / 17
GO:0044421
extracellular region...
0.411
3 / 82
GO:0044423
virion part
0.411
1 / 17
GO:0031012
extracellular matrix
0.353
3 / 58
GO:0019028
viral capsid
0.411
1 / 11
GO:0005578
proteinaceous extrac...
0.411
1 / 12
